# Mr. Pearson, on Cold Affusion in Typhus

**Published:** 1802-10-01

**Authors:** John Pearson

**Affiliations:** No. 15, Thornhaugh Street, Bedford Square


### *To the Editors of the Medical and Phyfical Journal.*

Gentlemen,

I Felt great pleafure on reading Mr. Blegborough's Obfervations on the ufe of cold Water in the treatment of Typhus, in your valuable Journal of Auguft laft. My

My mind fully enters into, and goes along with every sentiment there expressed; and I trust I may, without presumption, be allowed an opinion on this occasion, having within the last six months, as he expresses it, passed the fiery ordeal myself. In my case, the treatment recommended by Mr. Blegborough was adhered to exactly, and under his own direction, so that the conviction of its propriety may be rather said to have obtruded itself upon me, than to have been sought for; and I am at a loss to know which to admire most, the simplicity, or the comfort and the advantage of the plan. That the use of cold Water in Typhus should not have become more general amongst medical men, since the writings of Dr. Currie, is something astonishing; and more so, since I have experienced the beneficial effects of it myself. Amongst the reasons, perhaps, a powerful one is, that physicians, and more particularly the eminent, are seldom called at the commencement of the disease, so that they have but few opportunities of noting its progress, which is a material circumstance in the treatment of a disease, the propriety of which appears to consist more in preventing the accumulation of those symptoms that go to the composition of its worst form, than in curing them, after they have been suffered to take place; unless we wish to create giants, with no other intention than that we may have the satisfaction of destroying them, without regard to the hazard of the conflict. It has been confidently advanced by one physician, that Typhus was no longer formidable, since it might, at any time, be safely and expeditiously cured by a table spoonful of yeast. Though this gentleman has called it a specific, he has, nevertheless, never made good his position; and I cannot understand how it can act, except by the antiseptic quality of the fixed air which it contains: The antiseptic and tonic quality of bark are now, I believe, generally allowed to depend upon the tanning principle that enters into its composition.

The necessity of these, however, as well as all the more powerful antiseptics, is entirely superseded by the use of cold bathing: By the timely application of this remedy, the stomach, which should in every stage of the disease be as little as possible interfered with, is spared the trouble of assimilating what, under other circumstances, might be necessary, but what the system always struggles to reject; and thus is a conflict suspended, which by inducing heat, often lays the foundation of all the subsequent mischief.

In a matter of so much importance as the treatment of Typhus Fever, nothing ought to be assumed, but facts ought carefully to be noted as they occur. That the application of cold water is followed by the happiest effects, in the cure of this disease, is a very important one; and I have no doubt, and trust ere

 long, long, to fee it univerfally adopted to the full extent of its utility. After the full, clear, and perfpicuous account which Mr. Blegborough has given of the matter, it would be prefumptuous in me, to enter more fully into it; neither have I vanity enough to fuppofe, that I am able to add any thing of confequence to his account. Thus much I have confidered it my duty to ftate; and I fhall be very happy indeed, if my teftimony may be found to add weight to the propagation of a plan, which I am confident is founded in reafon, and will be fupported by experience, for there is not the fmalleft doubt, and I have no hefitation in faying, from my own knowledge of my cafe, that the more it is put in practice, and followed up, the more it will be admired. I am, &c.

*No.* 15, *Thornhaugh Street, Bedford Square,* JOHN PEARSON.

*September* 20, 1802.

---

